# A Grain-Based SARA Challenge Affects the Composition of Epimural and Mucosa-Associated Bacterial Communities throughout the Digestive Tract of Dairy Cows

**DOI:** 10.3390/ani11061658

**Published:** 2021-06-02

**Authors:** Jan C. Plaizier, Anne-Mette Danscher, Paula A. Azevedo, Hooman Derakhshani, Pia H. Andersen, Ehsan Khafipour

**Affiliations:** 1 Department of Animal Science, University of Manitoba, Winnipeg, MB R3T 2N2, Canada; Paula.Azevedo@umanitoba.ca (P.A.A.); derakhsh@mcmaster.ca (H.D.); 2 Department of Large Animal Sciences, Faculty of Health and Medical Sciences, University of Copenhagen, 2630 Taastrup, Denmark; amdanscher@gmail.com; 3 Department of Anatomy, Physiology and Biochemistry, Swedish University of Agricultural Sciences, 750-07 Uppsala, Sweden; pia.haubro.andersen@slu.se

**Keywords:** SARA, microbiota, dairy cows, digestive tract, MiSeq Illumina sequencing

## Abstract

**Simple Summary:**

High-yielding dairy cows must receive high-grain diets in order to meet their high energy requirements. However, these diets depress the pH in the rumen, leading to a condition referred to as subacute ruminal acidosis (SARA), and in the large intestine, and may negatively affect the taxonomic composition and the functionality of the populations of microorganisms in the digestive tract. As cows depend on these microorganisms for nutrient utilization and health, disruptions of their composition and functionality can greatly affect the production, health, and welfare of dairy cows. In our study, SARA was induced experimentally by excessive grain feeding. The taxonomic composition of bacterial populations attached to the epithelia of the digestive tract were determined throughout this tract. Our results show that SARA affected the populations of several taxa of bacteria, which suggests that the beneficial effects of these bacteria may be reduced, and that the digestive tract may be at increased risk of invasion by pathogenic microorganisms. The greatest effects of SARA on the taxonomic composition of bacteria on epithelia were in the rumen and large intestine. Their composition on epithelia in the small intestine was also affected, but the affected groups of bacteria differed from those in the rumen and large intestine.

**Abstract:**

The effects of a subacute ruminal acidosis (SARA) challenge on the composition of epimural and mucosa-associated bacterial communities throughout the digestive tract were determined in eight non-lactating Holstein cows. Treatments included feeding a control diet containing 19.6% dry matter (DM) starch and a SARA-challenge diet containing 33.3% DM starch for two days after a 4-day grain step-up. Subsequently, epithelial samples from the rumen and mucosa samples from the duodenum, proximal, middle and distal jejunum, ileum, cecum and colon were collected. Extracted DNA from these samples were analyzed using MiSeq Illumina sequencing of the V4 region of the 16S rRNA gene. Distinct clustering patterns for each diet existed for all sites. The SARA challenge decreased microbial diversity at all sites, with the exception of the middle jejunum. The SARA challenge also affected the relative abundances of several major phyla and genera at all sites but the magnitude of these effects differed among sites. In the rumen and colon, the largest effects were an increase in the relative abundance of Firmicutes and a reduction of Bacteroidetes. In the small intestine, the largest effect was an increase in the relative abundance of Actinobacteria. The grain-based SARA challenge conducted in this study did not only affect the composition and cause dysbiosis of epimural microbiota in the rumen, it also affected the mucosa-associated microbiota in the intestines. To assess the extent of this dysbiosis, its effects on the functionality of these microbiota must be determined in future.

## 1. Introduction

The high energy requirements of high-yielding dairy cows are commonly met by feeding them with high-grain diets. This can result in an accumulation of fermentation acids, including volatile fatty acids and lactate, in the rumen and the large intestine, and a reduction of rumen buffering, leading to depressions in the rumen and hindgut pH for extended periods each day [1,2,3]. These pH depressions contribute to gut health disorders, such as subacute ruminal acidosis (SARA) and hindgut acidosis [1,3,4]. These disorders affect the composition and functionality of microbiota and may result in microbiome dysbiosis and the establishment of opportunistic and pathogenic microorganisms in the digestive tract [3,5,6]. The symbiotic relationship between dairy cows and their gut microbiota is essential for the utilization of nutrients by these cows [7]. Hence, dysbiosis in the digestive tract can greatly affect their productivity and health. The rumen microbiota have been divided into fractions associated with the liquid digesta, the solid digesta, and the epithelium, also referred to as the epimural fraction [8,9,10]. The composition and functionality of the rumen epimural microbiota differs from those associated with digesta [8]. Especially, the epimural microbiota have a higher relative abundance of genera related to amino acid metabolism and a lower relative abundance of genes involved in carbohydrate metabolism [8]. The effects of high-grain feeding on epimural microbiota may therefore differ from those of digesta-associated microbiota. As epimural bacteria contribute to the barrier function and immune response of the epithelium [11], nutritional challenges such as SARA may increase the need for these contributions [3].

Recently, two meta-analyses have been conducted on the composition of ruminal epimural microbiota, determined by 16S rRNA sequencing [12,13]. Both studies concluded that many methodology-related factors, such as bioinformatic approaches, host species, geographic regions, diet, age, farm management practices, time of year, the hypervariable region sequenced, country of origin, farm, primer set, animal variability, and biopsy location affect the outcome of this analysis, but that conclusions across studies can be drawn. Anderson et al. [12] identified 147 core OTUs in cattle, including *Ruminococcus*, *Butyrivibrio*, other Lachnospiraceae, *Desulfobulbus*, *Desulfovibrio*, Neisseriaceae, and *Burkholderiaceae*, as well as the methanogenic archaea *Methanobrevibacter* and Methanomethylophilaceae. This study also concluded that, at the phylum level, epimural microbiota were dominated by Firmicutes, Bacteroidetes, and Proteobacteria, and that these accounted for 79.7% of total sequences. In their meta-analysis, Pacifico et al. [13] identified the bacterial taxa *Campylobacter*, the Christensenellaceae R-7 group, Defluviitaleaceae, UCG-011, Lachnospiraceae UCG-010, the Ruminococcaceae NK4A214 group, Ruminococcaceae UCG-010, Ruminococcaceae UCG-014, *Succiniclasticum*, *Desulfobulbus* and *Comamonas* spp. as the core taxa of rumen epimural microbiota in cattle. However, the definition of the core is a matter of debate [14].

Several studies on the effects of increased grain feeding and grain-induced SARA on epimural microbiota in the rumen have been conducted. Chen et al. [15] transitioned beef heifers from a 97% hay to an 8% hay diet, which reduced the relative abundances of the Proteobacteria and Bacteroidetes phyla, but did not affect that of the Firmicutes phylum. This transition also increased the relative abundances of the *Treponema*, *Ruminobacter* and Lachnospiraceae taxa. Wetzels et al. [16] determined the effects of 1-week and a 2-week grain-based SARA challenges separated by a 1-week break, during which an all forage diet was fed. They observed that the richness and diversity of epimural microbiota in the rumen were highest during the challenge break, and that more OTUs increased their relative abundance during the break than during the SARA challenges. A 4-week uninterrupted grain-based SARA challenge reduced the richness and diversity and the relative abundance of Proteobacteria, increased the relative abundance of Bacteroidetes, and did not affect that of Firmicutes in the ruminal epimural microbiota [17].

Studies on the composition and functionality of epimural microbiota in the small and large intestine are limited. However, Mao et al. [8] compared the composition of epimural microbiota across the digestive tracts of cattle, and observed three distinct clusters, including those in the forestomachs, the small intestine and the large intestine. They also reported that, across sites, the most prevalent phyla were Firmicutes, Bacteroidetes and Proteobacteria, but that the relative abundance of these phyla and of genera differed among the forestomachs, small intestine, and large intestine.

Based on the earlier research, we hypothesize that grain-based SARA challenges reduce the richness and diversity and alter the relative abundances of major phyla and genera of epimural and mucosa-associated microbiota in the small and large intestines, as well as that of the ruminal epimural microbiota, in dairy cows. Our main objective was therefore to characterize and compare the effects of a grain-based SARA challenge on the composition of epimural and mucosa-associated bacteria in the small and large intestines with those of the rumen in lactating dairy cows.

## 2. Materials and Methods

### 2.1. Animals and Sampling

Approval for the project was obtained from the Danish Animal Experiments Inspectorate (file no. 2012-15-2934-00052). Eight non-lactating rumen-cannulated Danish Holstein cows were fed a control diet with a forage-to-concentrate ratio of 83:17 (DM basis) for several weeks before the trial. The trial included four cows fed a control diet for two days, four days of gradually substituting 45% of the DM of the control diet with pellets containing 50% wheat and 50% barley, and two days on a full SARA diet (Table 1). The four control cows received the control diet throughout the study. More details on feed analysis, experimental design and methodology have been provided by Danscher et al. [18] and Plaizier et al. [19].

Cows were housed in individual tie stalls and had free access to fresh water. Cows were fed ad libitum, allowing for between 5% and 10% of feed refusals twice daily in equal portions at 8.00 AM and 14.30 PM. Reticular-rumen pH levels were monitored continuously using indwelling pH meter probes (eCow Rumen Analyzer, Exeter, United Kingdom), as described by Danscher et al. [18]. These probes were placed in the ventral sac of the rumen and were attached to 1 kg rounded stainless-steel weights in order to keep them positioned in the ventral sack. Their position was checked daily. The daily mean pH, as well as time below pH 5.8 and 5.6, were determined. Feces samples were obtained directly from the rectum at 15:00 and 21:00 on both SARA challenge days from all cows. The fecal pH was measured immediately after collection using a pH meter (Cardy Twin pH Meter, Spectrum Technologies Inc., Plainfield, IL, USA). A two-point calibration (pH 4 and 7) was performed before pH measurements.

All cows were euthanized after the SARA challenge by means of captive bolt stunning, followed by pithing and exsanguination at 2 h after morning feed delivery. Samples of 2 × 2 cm of whole tissue from the rumen wall, duodenum, proximal, middle and distal jejunum, ileum, cecum and colon were obtained with minimal handling. The rumen samples were obtained from the anterior ventral sac, immediately behind the ruminoreticular fold. The samples from the duodenum were obtained 15 cm distal to the pylorus. The proximal jejunum samples were obtained approximately 2 m aboral to the pylorus at the attachment to the mesentery vein. The middle jejunum samples were obtained midway between the pylorus and the ileocecal fold. The distal jejunum samples were obtained from the extended part of the mesentery, from the oral to the ileocecal fold. The samples from the ileum were collected midway between the ileocecal orifice and the ileocecal fold. The samples from the cecum and the colon were obtained from the free apex and the junction of the centripetal and centrifugal gyri, respectively. Samples were flushed gently with 0.9% saline, preserved in RNAlater (Ambion, Applied Biosystems, Foster City, CA, USA), snap frozen in liquid nitrogen and stored at −80 °C until downstream analysis.

### 2.2. DNA Extraction and MiSeq Illumina Sequencing

The tissue samples were removed from the −80 °C freezer one day prior to DNA extraction and thawed in a 4 °C refrigerator overnight. Following this, the samples were cryogenically homogenized using a Geno-Grinder 2010 (SPEX SamplePrep, Metuchen, NJ, USA). Subsequently, the DNA was extracted using a ZR-96 Fecal DNA Kit (Zymo Research, Irvine, CA, USA), which included a bead-beating step for the mechanical lysis of bacterial cells. Subsequently, DNA was eluted from the column with an elution buffer and the concentration and purity of the isolated DNA were assessed using a NanoDrop 2000 spectrophotometer (Thermo Scientific, Waltham, MA, USA). The DNA of all the samples was diluted with an elution buffer to a final nominal concentration of 20 ng/μL. The V4 region of the bacterial 16S rRNA gene was amplified and subjected to MiSeq Illumina sequencing. The reverse PCR primer was indexed with 12- base Golay barcodes, allowing for multiplexing of the samples.

The PCR reactions were conducted in duplicate and contained 1.0 μL of pre-normalized DNA, 1.0 μL each of forward and reverse primers (10 μM), 12 μL HPLC grade water (Fisher Scientific, Ottawa, ON, Canada) and 10 μL 5 Prime Hot MasterMix (5 Prime, Inc., Gaithersburg, MD, USA). Reactions consisted of an initial denaturing step at 94 °C for 3 min followed by 35 amplification cycles at 94 °C for 45 s, 50 °C for 60 s and 72 °C for 90 s; finalized by an extension step at 72 °C for 10 min in an Eppendorf Mastercycler Pro (Eppendorf, Hamburg, Germany). Following this, PCR products were purified using a ZR-96 DNA Clean-up Kit (ZYMO Research, Irvine, CA, USA) to remove primers, dNTPs and reaction components. The V4 library was generated by pooling 200 ng of each sample and quantified by PicogreendsDNA (Invitrogen, Grand Island, NY, USA). This was followed by multiple dilution steps, using pre-chilled hybridization buffer (HT1; Illumina, San Diego, CA, USA) to achieve pooled amplicon concentrations of 5 pM. Finally, 15% of PhiX control library was spiked into the amplicon pool to improve the unbalanced and biased base composition.

Customized sequencing primers for read1 (5t-TATGGTAATTGTGTGCCAGCMGCCGCGGTAA-3t), read2 (5t-AGTC AGTCAGCCGGACTACHVGGGTWTCTAAT-3t) and index read (5t-ATTAGAWACCCBDGTAGTCCG GCTGACTGACT3t) were synthesized and purified by polyacrylamide gel electrophoresis (Integrated DNA Technologies, Coralville, IA, USA). These primers were added to the MiSeq Reagent Kit V2 (300-cycle; Illumina, San Diego, CA, USA). The 150 paired-end sequencing reaction was performed on a MiSeq platform (Illumina, San Diego, CA, USA) at the Gut Microbiome Laboratory (Department of Animal Science, University of Manitoba, Winnipeg, MB, Canada).

### 2.3. Bioinformatics and Statistical Analyses

Overlapping paired-end reads were merged using the PANDAseq assembler [20]. All the sequences with low quality scores, as well as those containing uncalled bases (N) in the overlapping region, were discarded as described by Derakhshani et al. [21]. The subsequent merged reads were processed using QIIME v1.91 [22]. Briefly, merged reads were demultiplexed according to the barcode sequences and chimeric reads were filtered using UCHIME [23]. Reads were clustered into operational taxonomic units (OTU) based on 97% similarity using UCLUST [24]. Representative sequences from each OTU were assigned taxonomies using RDP Classifier [25], following alignment to the Greengenes reference database [26].

Standard alpha-diversity metrics, including the observed number of OTUs, Shannon index and Simpson index, were calculated at an even sequencing depth of 8000 per sample. In terms of beta-diversity analysis, weighted and unweighted UniFrac-based principal coordinates analysis (PCoA) were conducted with Phyloseq. Permutational multivariate analyses of variance (PERMANOVA) [27] based on the same similarity matrix was used to test the effects of the SARA challenge in each region of the digestive tract. The proportions of OTUs that were shared by cows were calculated for each treatment and each site of the digestive tract.

The effects of the SARA challenge, assessed by region, on the relative abundance data at the phylum and lower taxonomical levels were analyzed using a negative binomial test implemented in the DESeq2 package [28,29], according to a design containing treatment. Statistical differences were declared as significant at *p* < 0.05. Trends towards significance are discussed when *p* < 0.10.

The effects of the SARA challenge on the bacterial abundances and alpha-diversity indices were analyzed with SAS version 9.3 (SAS Institute, Cary, NC, USA). The UNIVARIATE procedure was used to test the normal distribution of variables and error terms. Nonnormally distributed variables were transformed using the Box–Cox power transformation implemented within TRANSREG procedure, which iteratively tests a variety of λ and identifies the best options. Normalized data were used to assess the effect of the fixed effect of treatment by region using the MIXED procedure of SAS version 9.3 (SAS Institute, Cary, NC, USA) with the SARA challenge as a fixed factor. For rumen pH and fecal pH, the effects of day, and in the case of fecal pH the fixed effects of time within day, were included in the model.

## 3. Results

The SARA challenge reduced the daily average rumen pH from 6.62 to 5.86 (*p* < 0.01) and the fecal pH from 6.7 to 5.0 (*p* < 0.05) (Table 2). The challenge reduced the minimum rumen pH from 6.09 to 5.19 (*p* < 0.05), without affecting the maximum rumen pH. This challenge also increased the durations for which the rumen pH remained below 5.8 and 5.6 from 2.0 to 653.2 min/d and from 0 to 486.6 min/d, respectively.

After trimming and quality control of the sequencing data, there were on average 30,631 ± 12,533 reads per sample for a total of 64 samples. Two samples, including a duodenum sample and a colon sample of SARA cows, were excluded because of low reads, i.e., <8000 reads per sample.

**Figure 1:**
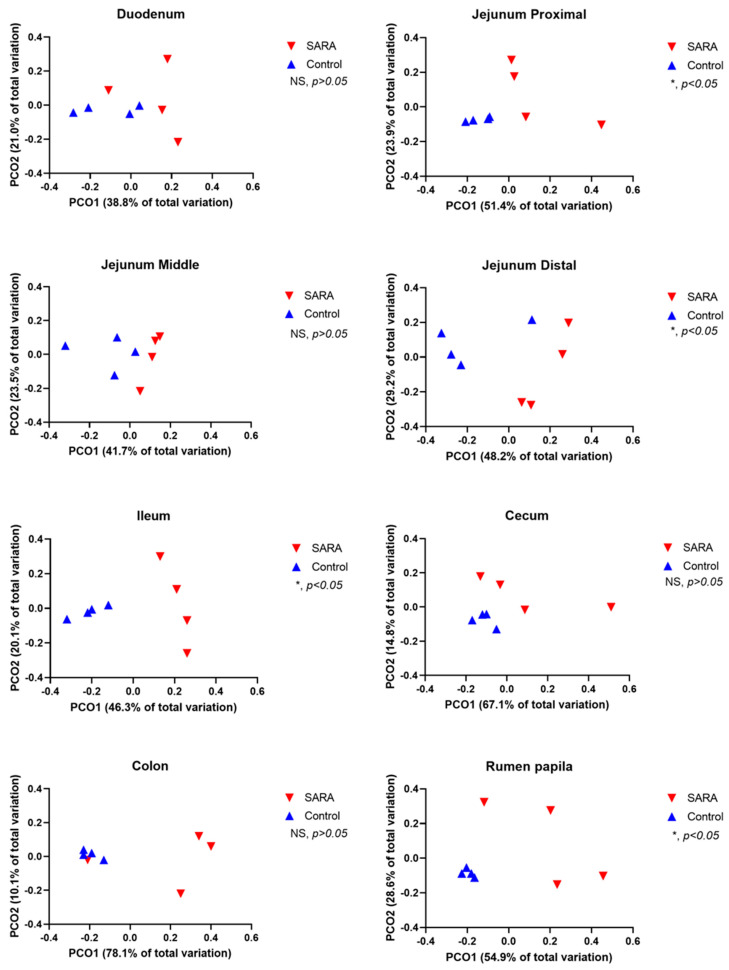
Principal coordinate analysis (PCoA) of unweighted UniFrac distances of treatments (C = control, S = SARA) by site in the digestive tract.

**Figure 2:**
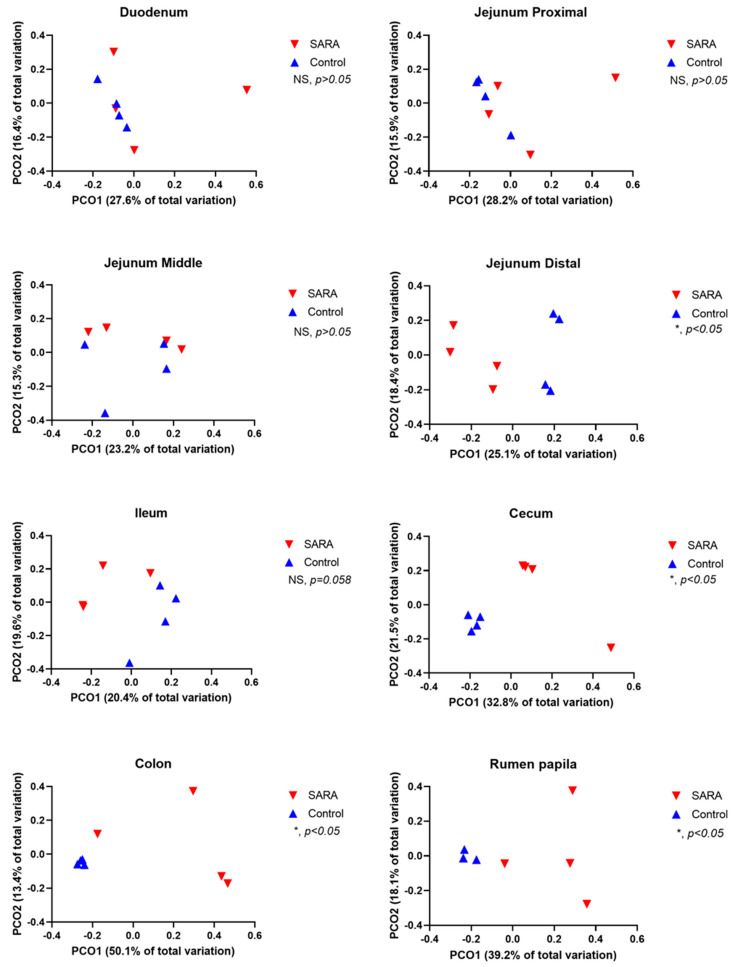
Principal coordinate analysis (PCoA) of weighted UniFrac distances of treatments (C = control, S = SARA) by site in the digestive tract.

The alpha-diversity varied among sites of the digestive tract with the rumen, cecum and colon having a higher richness and diversity than the small intestine (Table 3). The SARA challenge reduced the Shannon index at all sites with the exception of the middle jejunum (Table 3). This challenge also reduced the Chao 1 index in the rumen, cecum and colon. The observed number of OTUs was reduced by the challenge in the rumen, distal jejunum, ileum, cecum and colon. Both the weighted and the unweighted UniFrac PCoA analysis showed that the SARA challenge affected (*p* < 0.05) the composition of microbiota in the rumen, distal jejunum, cecum and colon (Figure 1 and Figure 2). A trend (*p* = 0.058) towards this effect existed in the ileum. The proportions of OTUs that were shared among cows, assessed by treatment and by site in the digestive tract, are shown in Venn diagrams in Supplementary Figure S1. For control cows, the percentages of shared OTUs were 30.5% for the rumen, 7.0% for the duodenum, 6.7% for the jejunum, 7.2% for the ileum, 26.4% for the cecum and 24.9% for the colon. For SARA cows, the percentages of shared OTUs were 4.8% for the rumen, 3.6% for the duodenum, 7.7% for the jejunum, 6.5% for the ileum, 3.7% for the cecum and 2.2% for the colon.

**Figure 3:**
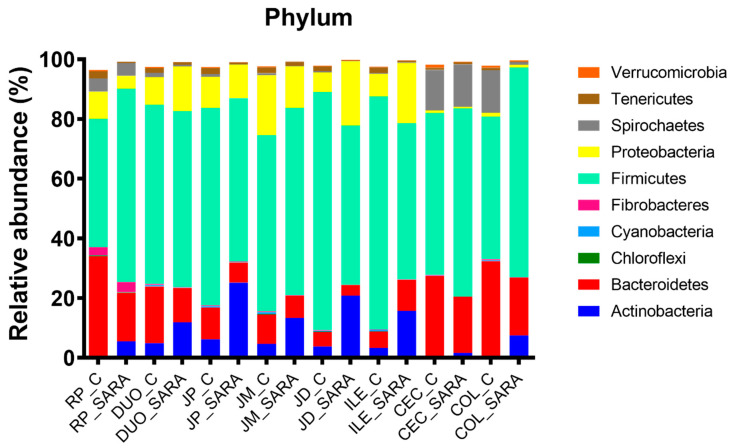
Relative abundances of abundant phyla, assessed by treatment (C = control, S = SARA), for each site in the digestive tract. RP = rumen, DUO = duodenum, JP = proximal jejunum, JM = middle jejunum, JD = distal jejunum, ILE = ileum, CEC = cecum, COL = colon.

Across treatments, the relative abundances of major phyla differed (*p* < 0.05) among sites (Figure 3). At the phylum level, the rumen, cecum and colon were dominated by Bacteroidetes and Firmicutes, whereas the duodenum, jejunum and ileum were dominated by Firmicutes, Bacteroidetes, Proteobacteria and Actinobacteria (Supplementary Table S1). Across the treatments, Spirochetes were more common in the cecum and colon than elsewhere in the digestive tract. The SARA challenge affected or tended to affect the relative abundances of eight out of 11 phyla in the rumen, five out of 11 phyla in the duodenum, six out of 11 phyla in the proximal jejunum, four out of 11 phyla in the middle jejunum, five out of 11 phyla in the distal jejunum, five out of 11 phyla in the ileum, five out of 11 phyla in the cecum and seven out of 11 phyla in the colon (Supplementary Table S1). In the rumen and the colon, the SARA challenge increased (*p* < 0.05) the relative abundance of Firmicutes and reduced (*p* < 0.05) that of Bacteroidetes. In contrast, this challenge did not affect these abundances in the cecum. In the rumen, cecum and colon, the SARA challenge reduced or tended to reduce the relative abundances of Proteobacteria and Cyanobacteria and increased that of Actinobacteria. The challenge reduced or tended to reduce the relative abundance of Verrucomicrobia in the rumen, duodenum and proximal jejunum, but tended to increase their abundance in the ileum. In the duodenum, jejunum and ileum, the SARA challenge increased or tended to increase the relative abundances of Actinobacteria and Cyanobacteria.

**Figure 4:**
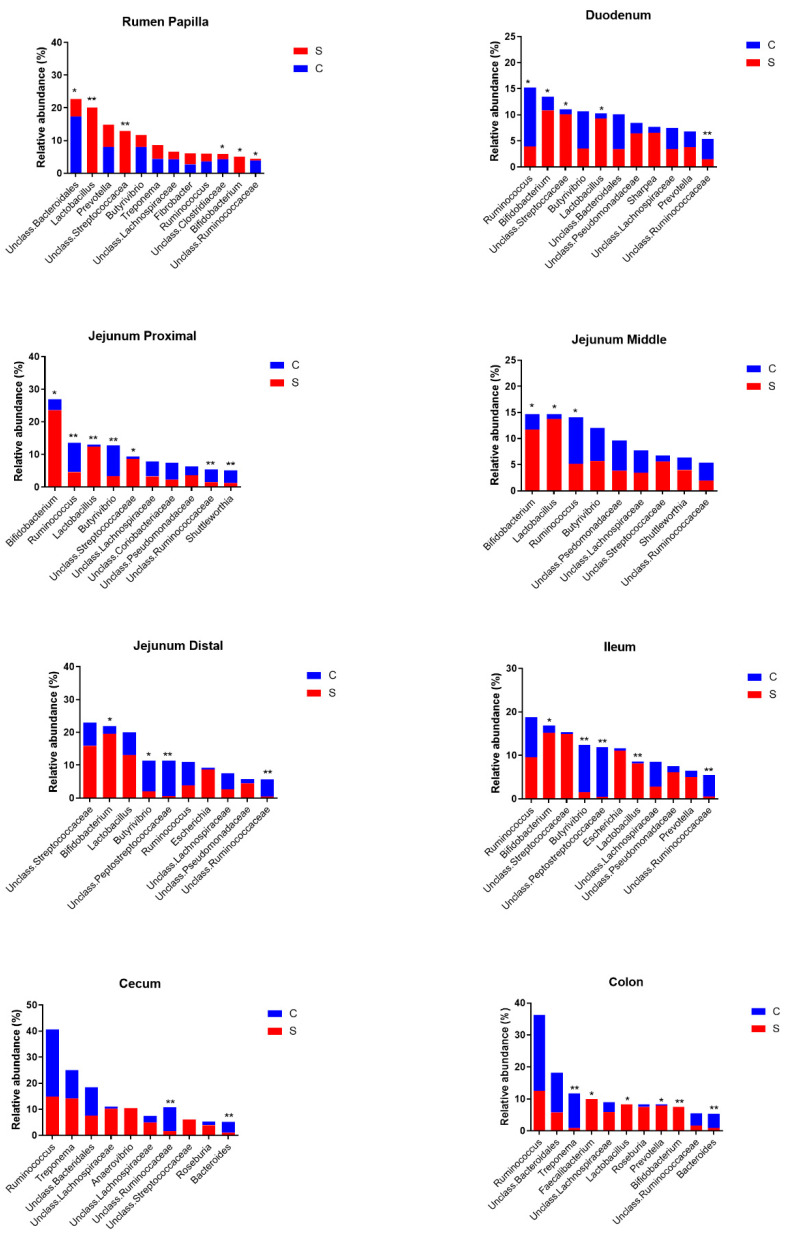
Effects of treatment (C = control, S = SARA) on the most abundant genera by site in the digestive tract. * Relative abundances of genera differ significantly (*p* < 0.05) among treatments, ** Relative abundances of genera differ significantly (*p* < 0.01) among treatments.

At the lowest taxonomical level, the most abundant taxa in the rumen during control or SARA experiments were unclassified Bacteroidales, *Lactobacillus*, *Prevotella*, unclassified Streptococcaceae, Butyrivibrio, *Treponema*, unclassified Lachnospiraceae, *Fibrobacter*, *Ruminococcus*, unclassified Clostridiaceae, *Bifidobacterium* and unclassified Ruminococcaceae (Figure 3 and Figure 4). Of these, the relative abundances of *Lactobacillus*, unclassified Streptococcaceae and *Bifidobacterium* were increased and the relative abundances of unclassified Bacteroidales, unclassified Lachnospiraceae, unclassified Clostridiaceae and unclassified Ruminococcaceae were decreased by the SARA challenge.

In the duodenum, the most abundant taxa were *Ruminococcus*, *Bifidobacterium*, unclassified Streptococcaceae, *Butyrivibrio*, *Lactobacillus*, unclassified Bacteroidales, unclassified Pseudomonaceae, *Sharpea*, unclassified Lachnospiraceae, *Prevotella* and unclassified Ruminococcaceae. Of these, the relative abundances of *Bifidobacterium*, unclassified Streptococcaceae and *Lactobacillus* increased and those of *Ruminococcus* and unclassified Ruminococcaceae decreased as a result of the SARA challenge.

The most abundant taxa in the proximal jejunum were *Bifidobacterium*, *Ruminococcus*, *Lactobacillus*, *Butyrivibrio*, unclassified Streptococcaceae, unclassified Lachnospiraceae, unclassified Coriobacteriaceae, unclassified Pseudomonadaceae, unclassified Ruminococcaceae and *Shuttleworthia*. Of these, the relative abundances of *Bifidobacterium*, *Lactobacillus* and unclassified Streptococcaceae increased and those of *Ruminococcus*, *Butyrivibrio*, unclassified Coriobacteriaceae, unclassified Ruminococcaceae and *Shuttleworthia* decreased as a result of the SARA challenge. In the middle jejunum, the most abundant taxa were *Bifidobacterium*, *Lactobacillus*, *Ruminococcus*, *Butyrovibrio*, unclassified Pseudomonadaceae, unclassified Lachnospiraceae, unclassified Streptococcaceae, *Shuttleworthia* and unclassified Ruminococcaceae. In this region, the SARA challenge increased the relative abundances of *Bifidobacterium* and *Lactobacillus* and decreased that of *Ruminococcus*. The most abundant taxa in the distal jejunum were unclassified Streptococcaceae, *Bifidobacterium*, *Lactobacillus*, *Butyrivibrio,* unclassified Peptostreptococcaceae, *Ruminococcus*, *Escherichia*, unclassified Lachnospiraceae, unclassified Pseudomonadaceae and unclassified Ruminococcaceae. Of these, the relative abundance of *Bifidobacterium* increased, and those of *Butyrivibrio*, unclassified Peptostreptococcaceae and unclassified Ruminococcaceae decreased as a result of the SARA challenge.

## 4. Discussion

In the current study, the SARA challenge resulted in a rumen pH depression of 486.6 min/d below pH 5.6 and 653.2 min/d below pH 5.8. We considered a rumen pH depression below 5.6 for more than 3 h/d and pH below 5.8 for more than 5–6 h/d as thresholds for SARA [30,31]. This shows that the SARA challenge caused a severe form of SARA. This challenge in our study also reduced the fecal pH from 6.7 to 5.9, showing that hindgut acidosis was also induced [3,4]. The parallel study by Danscher et al. [18] showed that the SARA challenge also reduced the feed intake and the concentration of acetate and the acetate to propionate ratio in the rumen. Danscher et al. [18] did not observe clinical signs of inflammation in SARA cows, but did report that the challenge decreased the calcium concentration and tended to increase the pCO2 levels of peripheral blood. This reduction in blood calcium was explained by the association between hypocalcaemia and SARA-induced endotoxemia. The increased pCO2 was seen as an indicator of an increased acid load on the bicarbonate buffer system. The parallel study by Plaizier et al. [19] showed that the SARA challenge reduced the richness and diversity of digesta-associated microbiota in the rumen and feces, and increased the relative abundance of Firmicutes in rumen digesta. These changes in the microbiota of rumen digesta and feces are commonly observed during grain-induced SARA [6,8,32,33]. The study by Plaizier et al. [19] also showed that the relative abundances of nine out of the 90 and 25 out of the 89 identified taxa in the rumen digesta and feces, respectively, were affected by the challenge. The results of Danscher et al. [18] and Plaizier et al. [19] are additional evidence for the successful induction of SARA and hindgut acidosis.

A comparison of the rumen and fecal pH depressions with those in earlier studies on SARA showed that the rumen and hindgut acidosis were more severe than in the studies of Tun et al. [32], Plaizier et al. [33] and Khalouei et al. [34]. This was expected due to the relatively high starch content of the SARA-challenge diet in the current study. In order to prevent acute ruminal acidosis in the current study, the SARA-challenge diet was only fed for two days. The induction of hindgut acidosis demonstrated that the SARA challenge increased fermentation in the hindgut, likely by increasing the amount of dietary starch that bypassed fermentation in the rumen and digestion in the small intestine [32,33,34]. The latter was demonstrated by Li et al. [35], who showed that a grain-based SARA challenge increased the starch content of the cecal digesta and feces from 4.2% to 6.1% and from 2.8% to 7.4% DM, respectively. This implies that the SARA challenge also increased the starch content of digesta in the small intestine. Hence, the SARA challenge did not only affect the environment for microbiota in the rumen, but also in the small and large intestine.

The effects of excessive grain feeding resulting in SARA on the composition of digesta-associated microbiota in the digestive tract of cattle has been studied extensively. These studies agree that these microbiota cluster according to the site in the digestive tract and to the level of grain feeding, and that three different site clusters—i.e., the rumen, the jejunum/ileum and the cecum/colon/rectum—exist [8,36,37]. In agreement with our results, Mao et al. [8] also found that epimural microbiota clustered according to the site in the digestive tract and to the level of grain feeding. This clustering may be explained by differences in pH, concentrations of VFA, the depth of the loosely adherent mucus layer and the differing composition of immune cells and functionality of the immune system among different sites in the digestive tract post-rumen [8]. In our study, SARA cows did not cluster together as closely as control cows. This indicates that the dysbiosis caused by the grain feeding depended on the individual cow [12,13].

Furthermore, in agreement with earlier studies, the SARA challenge reduced the richness and diversity of epimural microbiota in the rumen [8,15,16,17]. In addition, we observed that the challenge also reduced this richness and diversity in the small and large intestine, with the exception of the middle jejunum. Hence, although increased grain feeding increases the availability of substrates for bacterial fermentation, only opportunistic faster-growing microorganisms can take advantage of this condition [7,38,39]. This makes the environment in the digestive tract unfavorable for many other microorganisms, thereby reducing their populations and the overall richness and diversity of microbial communities throughout the digestive tract [11,40]. Such reductions in richness and diversity are perhaps undesirable, as they can reduce the functionality, resilience and robustness of these microbiota [39,40]. However, as different members of microbiota can share functionality and functional redundancy exists, a reduction in microbial richness and diversity may not always translate to reduced functionality [38,39]. The comparison of the proportions of shared OTUs showed that for control cows, the proportion of OTUs that are shared in the small intestine are low compared to those in the rumen and the large intestine. In SARA cows, the percentages of shared OTUs in the rumen, cecum and colon were much lower than in control cows. These reductions may be due to the drop in the digesta pH in these sites and the resulting dysbiosis.

In our study, the relative abundances of major phyla of epimural microbiota differed among sites. At the phylum level, the rumen, cecum and colon were dominated by Firmicutes (46.4%), Bacteroidetes (24.6%), Spirochetes (8.6%), Proteobacteria (4.9%) and Spirochetes (8.6%). At this level, the small intestine was dominated by Firmicutes (62.5%), Bacteroidetes (8.6%), Proteobacteria (10.8%) and Actinobacteria (10.7%). Our results on phyla in the ruminal epimural microbiota are generally in agreement with the metanalyses of Anderson et al. [12] and Pacifico et al. [13], with the exception that in their metanalysis Proteobacteria were more abundant and Actinobacteria were less abundant. Mao et al. [8] reported that, although Firmicutes was the most abundant phylum on mucosa-associated microbiota in most sites of the digestive tract, the second most abundant phylum differed among regions. They reported that Bacteroidetes was the second most abundant mucosa-associated phylum in the reticulum, omasum, abomasum, colon and rectum, whereas Proteobacteria was the second most abundant in the jejunum and ileum, and the most abundant mucosa-associated phylum in the duodenum. In addition, both Firmicutes and Spirochaetes were the second most dominant mucosa-associated phyla in the duodenum and cecum. Our results are mostly in agreement with those of Mao et al. [8], including that the relative abundances of Proteobacteria were higher in the small intestine than in the rumen and large intestine.

Studies on the effects of grain feeding on epimural microbiota have mainly concentrated on the rumen, and results have varied among these studies. In our study, the SARA challenge reduced the relative abundance of Proteobacteria and Bacteroidetes, but increased that of Firmicutes in the rumen. In contrast, transitioning beef heifers from a 97% forage diet to a 92% concentrate diet reduced the relative abundances of Firmicutes and Proteobacteria in the ruminal epimural microbiota [15]. In addition, in contrast with our findings, Mao et al. [8] reported that the relative abundance of Firmicutes was not affected by the SARA challenge in the proximal part of the jejunum and in the cecum, but this abundance was reduced in the distal jejunum, and in the ileum this challenge reduced this abundance. The effects of the SARA challenge on the relative abundances of epimural phyla in the large intestine were similar to those in the rumen, in that there was an increase in the relative abundance of Firmicutes and a decrease in those of Bacteroides and Proteobacteria. The increase in the relative abundance of Actinobacteria that occurred in the small intestine was not observed in the rumen.

In the current study, the most abundant epimural genera varied among sites of the digestive tract. In the rumen, they included unclassified Bacteroidales, *Lactobacillus*, unclassified Lachnospiraceae, unclassified Clostridiaceae, unclassified Ruminococcaceae, unclassified Streptococcaceae, *Butyrivibrio* and *Prevotella*. In the small intestine, these included *Ruminococcus*, *Bifidobacterium*, *Lactobacillus*, unclassified Streptococcaceae and *Butyrivibrio*, whereas in the large intestine these included *Ruminococcus*, unclassified Bacteroidales and *Treponema*. A metanalysis of the ruminal epimural microbiota in ruminants demonstrated that abundant taxa include *Ruminococcus*, *Butyrivibrio*, other Lachnospiraceae, *Desulfobulbus*, *Desulfovibrio*, Neisseriaceae and Burkholderiaceae, as well as methanogenic archaea *Methanobrevibacter* and Methanomethylophilaceae [12]. Another such metanalysis identified *Campylobacter*, Christensenellaceae R-7 group, Defluviitaleaceae, UCG-011, Lachnospiraceae UCG-010, Ruminococcaceae NK4A214 group, Ruminococcaceae UCG-010, Ruminococcaceae UCG-014, *Succiniclasticum*, *Desulfobulbus* and *Comamonas* spp. as core taxa of the ruminal epimural microbiota [13]. Hence, our results agree with these metanalyses that unclassified Lachnospiraceae, unclassified Ruminococcaceae and *Butyrivibrio* are abundant in the ruminal epimural microbiota, but the other abundant genera identified in the metanalyses were not prominent in our study. In agreement with our findings, *Butyrivibrio* and *Lactobacillus* were present in the epimural ruminal microbiota in a study by Petri et al. [41], although *Lactobacillus* was only detected in cows on an acidotic challenge diet.

Our findings also agree with Mao et al. [8] in that unclassified Bacteroidales were more abundant in the epimural microbiota of the rumen and large intestine than in the small intestine. Furthermore, these authors observed that Prevotellaceae were most abundant in the epimural microbiota of the rumen and *Treponema* was most abundant in the microbiota of the large intestine. However, in contrast to our study, *Lactobacillus* was not prevalent in the epimural microbiota of the study of Mao et al. [8]. Hence, many of the abundant epimural ruminal taxa in our study were not abundant in the metanalyses of the epimural ruminal microbiota conducted by Anderson et al. [12] and Pacifico et al. [13]. This confirms the conclusion of these meta-analyses that many methodology-related and non-dietary factors affect the outcome of the taxonomic analysis of epimural microbiota. However, due to the multitude of these factors, it is not possible to determine which of them were responsible for the differences between our results and those of previous studies.

In our study, seven genera of epimural microbiota were affected by the SARA challenge. In agreement with our study, Petri et al. [41] showed that SARA increased the relative abundance of *Lactobacillus* in the ruminal epimural microbiota. However, the increases in the relative abundances in *Atopobium*, *Desulfocurvus*, *Fervidicola*, *Solobacterium*, *Succinivibrio*, *Olsenella*, *Succiniclasticum*, *Roseburia* and *Sharpea* that were reported by Petri et al. [41] were not observed in our study. In addition, decreases in the relative abundances of *Kingella*, *Azoarcus*, *Altererythrobacter*, *Alkalibaculum*, *Acidaminobacter*, *Oscilibacter*, *Saccharofermentans*, *Lurtispora*, *Fastidiospila*, *Tannerella*, *Howardella*, *Bifidobacterium*, *Kinoniella*, *Acetonema*, *Sutrerella*, *Anaplasma*, *Siphonobacter* and *Desulfanatronum*, and increases in the abundance of *Coprobacillus*, due to an intermittent SARA challenge conducted by Wetzels et al. [16] were not evident in our study. In addition, reductions in the relative abundances of *Kingella*, *Saccharofermentans*, *Lutispora*, *Azospira*, *Pseudosphingobacterium*, *Coprobacillus*, *Thioreductor*, *Syntrophococcus*, *Anaerorhabdus*, *Brevinema* and *Petrimonas* in these microbiota, as well as increases in the abundances of *Anaerophagam*, *Succiniclasticum*, *Fastidiospila* and *Selenomonas* resulting from a 4-week SARA challenge conducted Wetzels et al. [17] did not occur in our study.

Our study showed that the relative abundances of five, eight, three, four and five taxa in, respectively, the duodenum, proximal jejunum, middle jejunum, distal jejunum and ileum were affected by the SARA challenge. In the cecum and colon, the relative abundances of three and five taxa, respectively, were affected by this challenge. Across the sites of the digestive tract, the largest increases in relative abundances were observed for *Lactobacillus* and *Bifidobacterium*, whereas Ruminococcaceae and *Butyrivibrio* showed the largest decreases.

A comparison of our study with earlier studies on the effect of grain-feeding on the epimural ruminal microbiota shows that these studies agree that a grain-based SARA challenge reduces the richness and diversity of these microbiota, but that the effects of such a challenge on the abundances of abundant phyla and genera are highly variable. Anderson et al. [12] and Pacifico et al. [13] suggested that this variation is the result of various differences in experimental and analytical methodologies among studies. Our study has also shown that a grain-based SARA challenge also reduces the richness and diversity of epimural microbiota in the small and large intestine. Reductions in the richness and diversity of microbiota can be considered to constitute dysbiosis [38,39]. However, such reductions may also reflect a non-pathological adaptation to a change in diet, and changes in the functionality of microbiota [39,40,41]. Hence, in order to asses if reductions in microbial richness and diversity constitute dysbiosis, the change in the functionality of the microbiota needs to be determined. A key consideration in this regard is the concept of influential members—the keystone and foundation members of microbiota [42]—that are the drivers of the community. Changes in the diversity or the abundances of major phyla and taxa would not affect the functionality of microbial community as long as its influential members were able to maintain their populations and abundances. Hence, determining the effects of dietary changes on the functionality of gut microbiota may be more important than determining the changes in their taxonomic composition.

Diet-induced changes in the composition of epimural microbiota have also been associated with reductions of the barrier function of epithelia in the digestive tract [9,43,44]. This has been linked to systemic inflammation and inflammatory bowel disease in humans, and to liver abscesses, laminitis and transient aseptic synovitis in cattle, and may be caused by translocation of a variety of microorganisms or their toxins out of the digestive tract [3,42,45]. Alternately, antibodies against rumen bacteria have been detected in colostrum and milk, and may have health benefits for the calf [46]. This implies that maintaining diverse and functioning epimural microbiota is key to the overall health and gut health of cattle.

## 5. Conclusions

A short-term SARA challenge resulted in severe forms of SARA and hindgut acidosis, affected the taxonomic composition and reduced the microbial richness and diversity of epimural and mucosa-associated microbiota throughout the digestive tract of non-lactating dairy cows. This suggests that this challenge caused dysbiosis of these microbiota throughout the digestive tract. The site of the digestive tract affected the composition of the microbiota, as three distinct clusters of microbiota were observed, i.e., the rumen, the jejunum/ileum and the cecum/colon. The relative abundances of several major phyla and genera of epimural and mucosa-associated microbiota were affected at all sites. In the rumen and large intestine, the largest effect observed was an increase in the Firmicutes-to-Bacteroidetes ratio. In the small intestine, the largest effect observed was an increase in the relative abundance of Actinobacteria, including members of *Bifidobacterium*. The relative abundances of a small proportion of abundant taxa were affected by the SARA challenge, but these effects varied among sites.

## Figures and Tables

**Table 1:** Ingredient and chemical composition of the experimental diets.

	Control Diet	SARA Diet
Ingredient composition, % DM		
Total mixed ration (TMR) ^1^	100	55
Wheat/barley pellets ^2^	0	45
Chemical composition		
Dry matter, %	54.7	61.5
Crude protein, % DM	11.3	11.0
Neutral detergent fiber, % DM	53.4	35.0
Acid detergent fiber, % DM	30.5	19.9
Starch, % DM	14.8	30.7
Calcium % DM	0.57	0.34
Phosphorus, %DM	0.29	0.30
Magnesium, % DM	0.18	0.15
Potassium, % DM	1.77	1.18
Sodium, % DM	0.19	0.11

^1^ TMR contains barley straw (27.9% DM), rapeseed meal (9.7% DM), vitamin/mineral mix (1.1% DM), beet pulp (5.4% DM), grass silage (20.8% DM) and corn silage (34.7% DM). ^2^ Wheat barley pellets contain 50% DM ground wheat and 50% DM ground barley.

**Table 2:** Rumen and fecal pH of cows on the control and the SARA-challenge diet.

Parameter	Treatment		SE	Significance
	Control	SARA		*p*-Values
Avg. rumen pH	6.62 ^a^	5.86 ^b^	0.13	<0.01
Time rumen pH < 5.8, min/d	3.0 ^b^	662 ^a^	107.7	<0.01
Time rumen pH < 5.6, min/d	0 ^b^	493 ^a^	93.6	<0.01
Minimum rumen pH	6.09	5.19	0.22	<0.01
Maximum rumen pH	6.73	6.90	0.10	0.18
Fecal pH	6.7 ^a^	5.9 ^b^	0.18	<0.05

^a, b^ LSmeans with different superscripts in a row differ (*p* < 0.05).

**Table 3:** Effects of a grain-based SARA challenge (SARA) on bacterial richness and diversity indices throughout the digestive tracts of dairy cows.

Site	Shannon			Chao1			Observed OTU		
	Control	SARA	*p*-Value	Control	SARA	*p*-Value	Control	SARA	*p*-Value
Rumen	8.74 ^a^	6.09 ^b^	0.04	2990.8 ^a^	1197.2 ^b^	0.002	1603 ^a^	733 ^b^	0.003
Duodenum	8.24 ^x^	6.37 ^y^	0.05	1048.8	837.9	0.69	837	617	0.32
Jejunum proximal	7.96 ^a^	5.77 ^b^	0.04	888.2	577	0.11	720	465	0.11
Jejunum middle	7.52	6.55	0.22	611.9	674.6	0.89	509	525	0.92
Jejunum distal	7.01 ^a^	4.79 ^b^	0.04	805.1 ^x^	492.3 ^y^	0.06	618 ^a^	345 ^b^	0.01
Ileum	7.61 ^a^	4.98 ^b^	0.003	999.08	712.8	0.11	766 ^a^	502 ^a^	0.01
Cecum	8.46 ^a^	5.77 ^b^	0.0	2665.7 ^a^	1237.8 ^b^	0.005	1468 ^a^	722 ^b^	0.003
Colon	8.48 ^a^	6.10 ^b^	0.02	2405.0 ^a^	994.3 ^b^	0.004	1388 ^a^	603 ^b^	0.005

^a, b^ LSmeans with different superscripts in a row differ (*p* < 0.05); ^x, y^ LSmeans with different superscripts in a row tend to differ (*p* < 0.10).

## Data Availability

All sequencing data were deposited as NCBI BioProject record ID# PRJNA738809 and can be accessed though URL: https://www.ncbi.nlm.nih.gov/bioproject/?term=PRJNA738809.

## Supplementary Materials

The following are available online at https://www.mdpi.com/article/10.3390/ani11061658/s1, Table S1: Effects of the SARA challenge on the relative abundances of phyla with an abundance above 0.1% of community, assessed by site of the digestive tract. Supplementary Figure S1A–K. Venn diagrams of shared OTUs in epimural microbiota.

## Abbreviations

ADF	Acid detergent fiber
CP	Crude protein
DM	Dry matter
NDF	Neutral detergent fiber
OTU	Operational taxonomic unit
PCR	Polymerase chain reaction
SARA	Subacute ruminal acidosis
