# Supplementary material for: A Grain-Based SARA Challenge Affects the Composition of Epimural and Mucosa-Associated Bacterial Communities throughout the Digestive Tract of Dairy Cows
Source: Animals (Basel). 2021 Jun 2;11(6):1658. doi: 10.3390/ani11061658 (PMC8227306; doi:10.3390/ani11061658)
Supplement: Supplementary file 1 [file animals-11-01658-s001.zip › animals-1182723-supplementary for update.pdf]

Table S1. Effects of the SARA challenge on the relative abundances of phyla with an abundance above 0.1% by region of the digestive tract.

		**Rumen**		
	**Control**	**SARA**	**SEM**	**Effects, *P values***
Firmicutes	43.0b	64.8a	10.2	0.05
Bacteroidetes	33.7a	16.2b	5.69	0.05
Proteobacteria	9.0x	4.2y	2.32	0.08
Spirochaetes	4.5	4.2	3.02	0.21
Fibrobacteres	2.7	3.4	2.46	0.29
Tenericutes	2.4a	0.5b	0.77	0.005
Euryarchaeota	0.79a	0.24b	0.096	0.01
Verrucomicrobia	0.36a	0.08b	0.037	0.002
Actinobacteria	0.31b	5.54a	1.34	0.005
Chloroflexi	0.30x	0.13y	0.056	0.08
Cyanobacteria	0.029	0.13	0.041	0.13
		**Duodenum**		
	**Control**	**SARA**	**SEM**	**Effects, *P values***
Firmicutes	60.00	59.00	7.08	0.88
Bacteroidetes	18.90	11.50	4.76	0.32
Proteobacteria	9.34	14.90	5.93	0.88
Actinobacteria	4.90y	11.90x	2.34	0.06
Euryarchaeota	1.60a	0.32b	0.39	0.04
Tenericutes	1.57	0.90	0.43	0.27
Spirochaetes	1.41	0.53	0.71	0.22
Cyanobacteria	0.52a	0.07b	0.13	0.02
Verrucomicrobia	0.43a	0.05b	0.11	0.02
Fibrobacteres	0.33a	0.12b	0.06	0.03
Chloroflexi	0.07	0.07	0.05	0.96
		**Proximal jejunum**		
	**Control**	**SARA**	**SEM**	**Effects, *P values***
Firmicutes	66.1	54.7	8.43	0.42
Bacteroidetes	10.6x	6.7y	1.23	0.07
Proteobacteria	10.4	11.2	2.18	0.85
Actinobacteria	6.2y	25.1x	7.78	0.07
Euryarchaeota	2.0a	0.4b	0.65	0.10
Tenericutes	2.0a	0.6b	0.37	0.03
Spirochaetes	0.92a	0.14b	0.143	0.005
Cyanobacteria	0.48x	0.14y	0.114	0.08
Verrucomicrobia	0.34x	0.14y	0.067	0.08
Fibrobacteres	0.26	0.11	0.088	0.37
Chloroflexi	0.033	0.12	0.0522	0.31
		**Middle jejunum**		
	**Control**	**SARA**	**SEM**	**Effects, *P values***
Firmicutes	59.0	62.8	5.83	0.63
Proteobacteria	20.0	13.8	3.89	0.29
Bacteroidetes	9.8	7.3	1.69	0.33
Actinobacteria	4.7b	13.4a	2.91	0.05
Tenericutes	2.0	1.3	0.44	0.36
Euryarchaeota	1.40x	0.41y	0.37	0.08
Cyanobacteria	0.69x	0.10y	0.22	0.08
Spirochaetes	0.62	0.2	0.17	0.13
Verrucomicrobia	0.35	0.13	0.212	0.75
Fibrobacteres	0.18	0.062	0.078	0.34
Chloroflexi	0.17x	0.033y	0.044	0.05
		**Distal jejunum**		

	Control	SARA	SEM	Effects, *P values*
Firmicutes	79.8a	53.3b	6.62	0.03
Proteobacteria	6.5	21.5	5.01	0.13
Bacteroidetes	4.9	3.6	1.41	0.65
Actinobacteria	3.8b	21a	4.78	0.05
Euryarchaeota	1.7	0.12	0.36	0.005
Tenericutes	1.6	0.27	0.378	0.036
Spirochaetes	0.47	0.023	0.183	0.14
Cyanobacteria	0.32a	0.02b	0.074	0.02
Verrucomicrobia	0.21	0.11	0.077	0.26
Fibrobacteres	0.18	0.041	0.08	0.2
Chloroflexi	0.13a	0.025b	0.02	0.01
**Ileum**				
	**Control**	**SARA**	**SEM**	**Effects, *P values***
Firmicutes	78.0a	52.2b	7.97	0.05
Proteobacteria	7.8	20.2	7.79	0.95
Bacteroidetes	5.4	10.4	2.16	0.12
Actinobacteria	3.4b	15.8a	3.92	0.04
Euryarchaeota	2.0a	0.13b	0.306	0.01
Tenericutes	1.9x	0.6y	0.433	0.09
Cyanobacteria	0.51x	0.09y	0.17	0.09
Spirochaetes	0.28	0.15	0.088	0.39
Verrucomicrobia	0.23x	0.094y	0.053	0.10
Chloroflexi	0.13	0.026	0.05	0.18
Fibrobacteres	0.11	0.033	0.063	0.42
**Cecum**				
	**Control**	**SARA**	**SEM**	**Effects, P values**
Firmicutes	54.2	63.0	6.47	0.19
Bacteroidetes	27.2	18.8	5.15	0.12
Spirochaetes	13.6	14.2	5.93	0.82
Verrucomicrobia	0.95	0.16	0.40	0.13
Proteobacteria	0.86a	0.53b	0.079	0.02
Tenericutes	0.79	0.70	0.125	0.62
Euryarchaeota	0.37a	0.03b	0.047	0.01
Actinobacteria	0.20	1.70	0.54	0.05
Fibrobacteres	0.16a	0.004b	0.035	0.01
Cyanobacteria	0.15a	0.012b	0.044	0.03
Chloroflexi	0.011	0.004	0.0033	0.18
**Colon**				
	**Control**	**SARA**	**SEM**	**Effects, P values**
Firmicutes	47.8b	70.3a	5.38	0.02
Bacteroidetes	32.2a	19.4b	5.24	0.05
Spirochaetes	14.2a	0.88b	2.28	0.001
Proteobacteria	1.3	0.88	0.177	0.10
Tenericutes	0.83a	0.27b	0.157	0.04
Verrucomicrobia	0.66	0.37	0.29	0.23
Euryarchaeota	0.55a	0.06b	0.061	0.002
Cyanobacteria	0.35a	0.02b	0.074	0.006
Fibrobacteres	0.31a	0.00b	0.0356	0.0002
Actinobacteria	0.12b	7.50a	1.66	0.002
Chloroflexi	0.0068	0.0184	0.0077	0.34

^{a,b} Lsmeans with different superscripts in a row differ (P < 0.05), ^{x,y}Lsmeans with different superscripts in a row tend to differ (P <0.10).

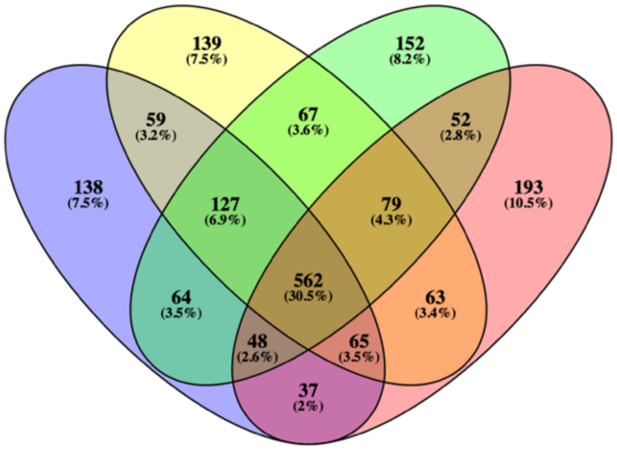

Supplementary figure 1A. Venn diagram of shared OTU in epimural microbiota in the rumen of control cows.

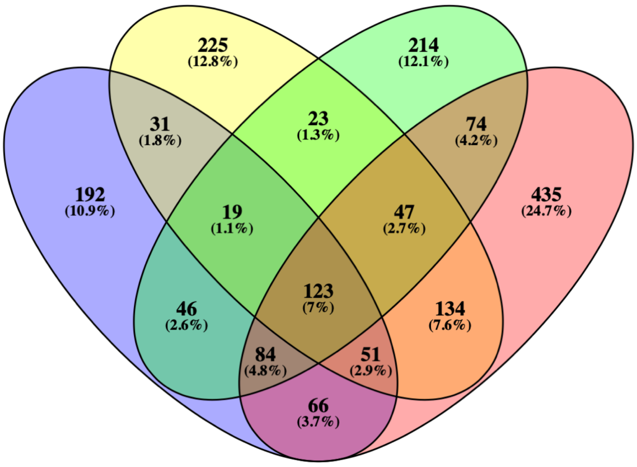

Supplementary figure 1B. Venn diagram of shared OTU in epimural microbiota in the duodenum of control cows.

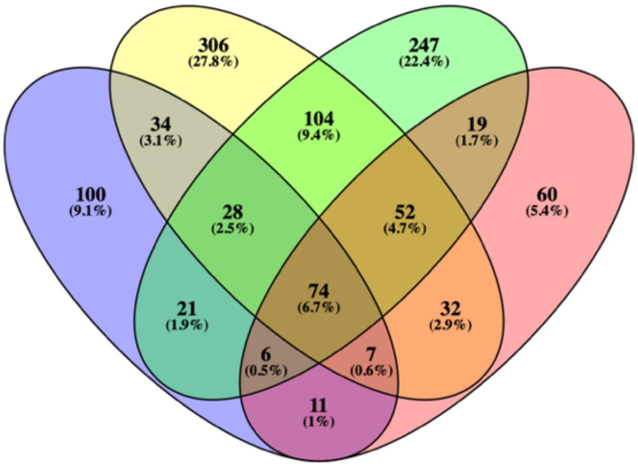

Supplementary figure 1C. Venn diagram of shared OTU in epimural microbiota in the jejenum of control cows.

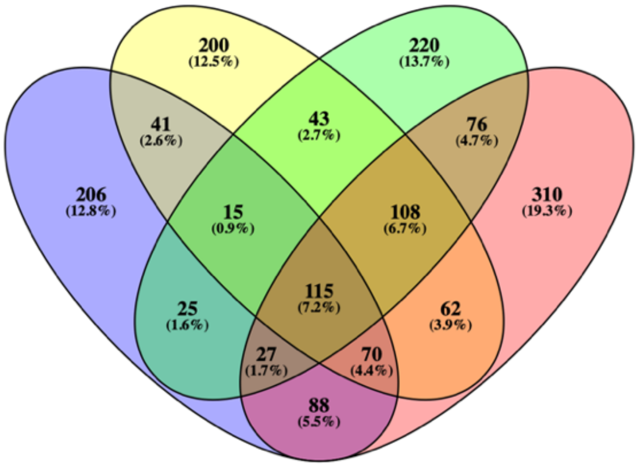

Supplementary figure 1D. Venn diagram of shared OTU in epimural microbiota in the ileum of control cows.

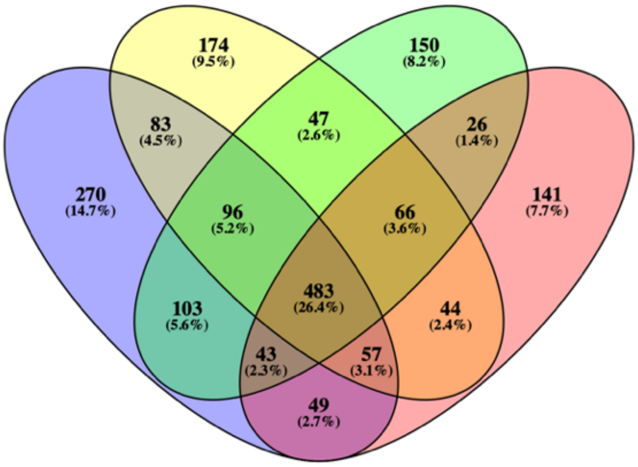

Supplementary figure 1E. Venn diagram of shared OTU in epimural microbiota in the cecum of control cows.

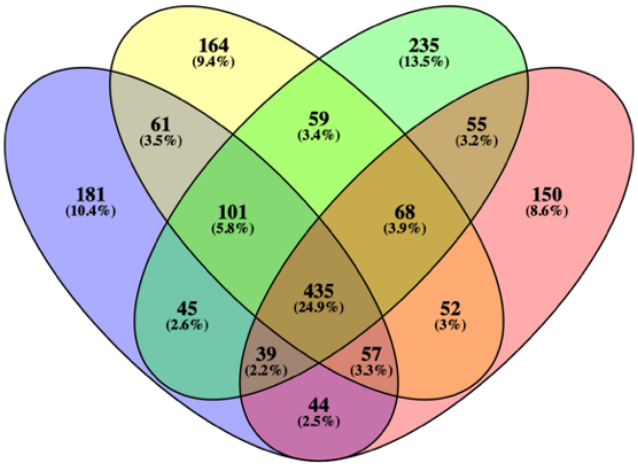

Supplementary figure 1E. Venn diagram of shared OTU in epimural microbiota in the colon of control cows.

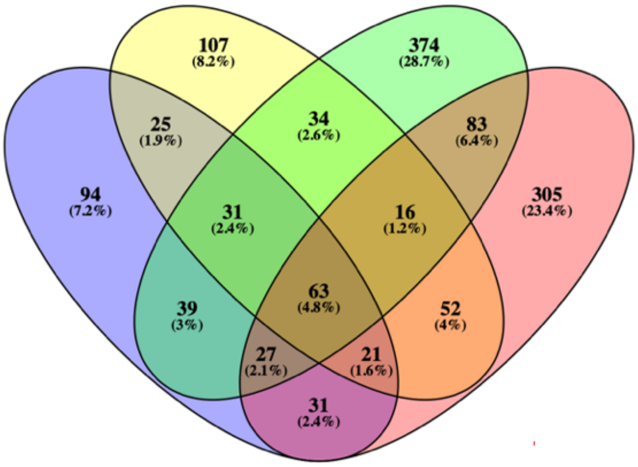

Supplementary figure 1F. Venn diagram of shared OTU in epimural microbiota in the rumen of SARA cows.

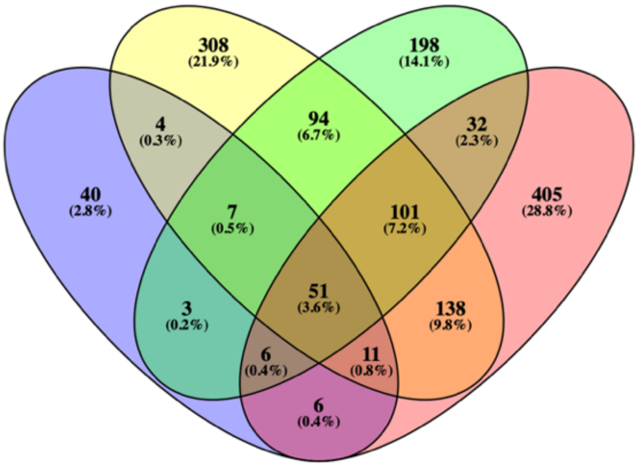

Supplementary figure 1G. Venn diagram of shared OTU in epimural microbiota in the duodenum of SARA cows.

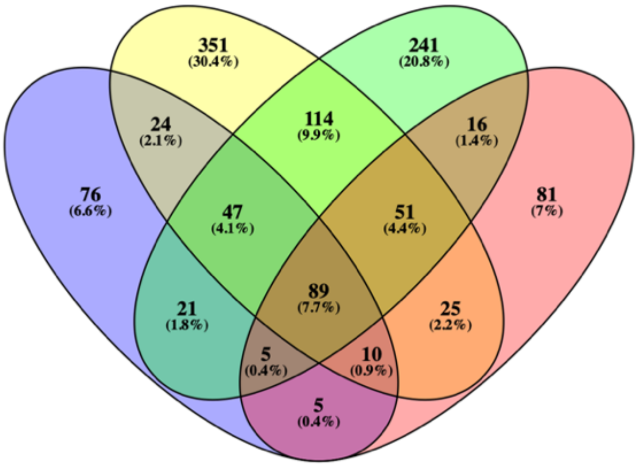

Supplementary figure 1H. Venn diagram of shared OTU in epimural microbiota in the jejunum of SARA cows.

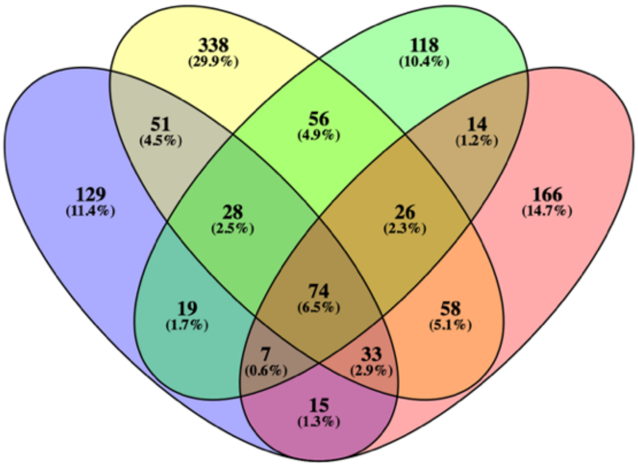

Supplementary figure 1I. Venn diagram of shared OTU in epimural microbiota in the ileum of SARA cows.

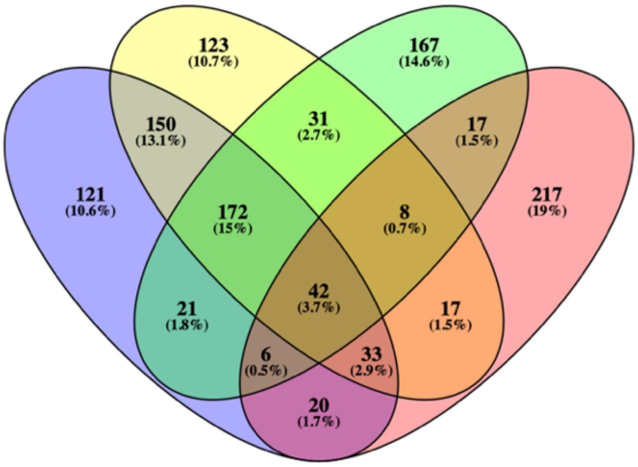

Supplementary figure 1J. Venn diagram of shared OTU in epimural microbiota in the cecum of SARA cows.

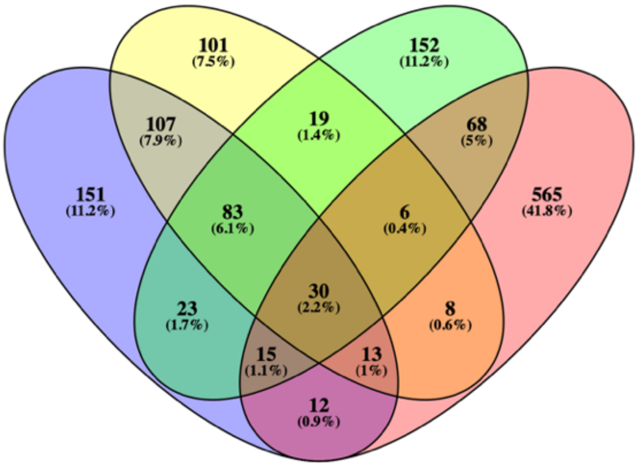

Supplementary figure 1K. Venn diagram of shared OTU in epimural microbiota in the colon of SARA cows.
